# Supplementary material for: Seminoma and Embryonal Carcinoma Footprints Identified by Analysis of Integrated Genome-Wide Epigenetic and Expression Profiles of Germ Cell Cancer Cell Lines
Source: PLoS One. 2014 Jun 2;9(6):e98330. doi: 10.1371/journal.pone.0098330 (PMC4041891; doi:10.1371/journal.pone.0098330)
Supplement: File S1 — ZIP file containing DMRforPairs output for significant regions. Please start from the html files. (ZIP) [file pone.0098330.s008.zip › figures/14183.pdf]

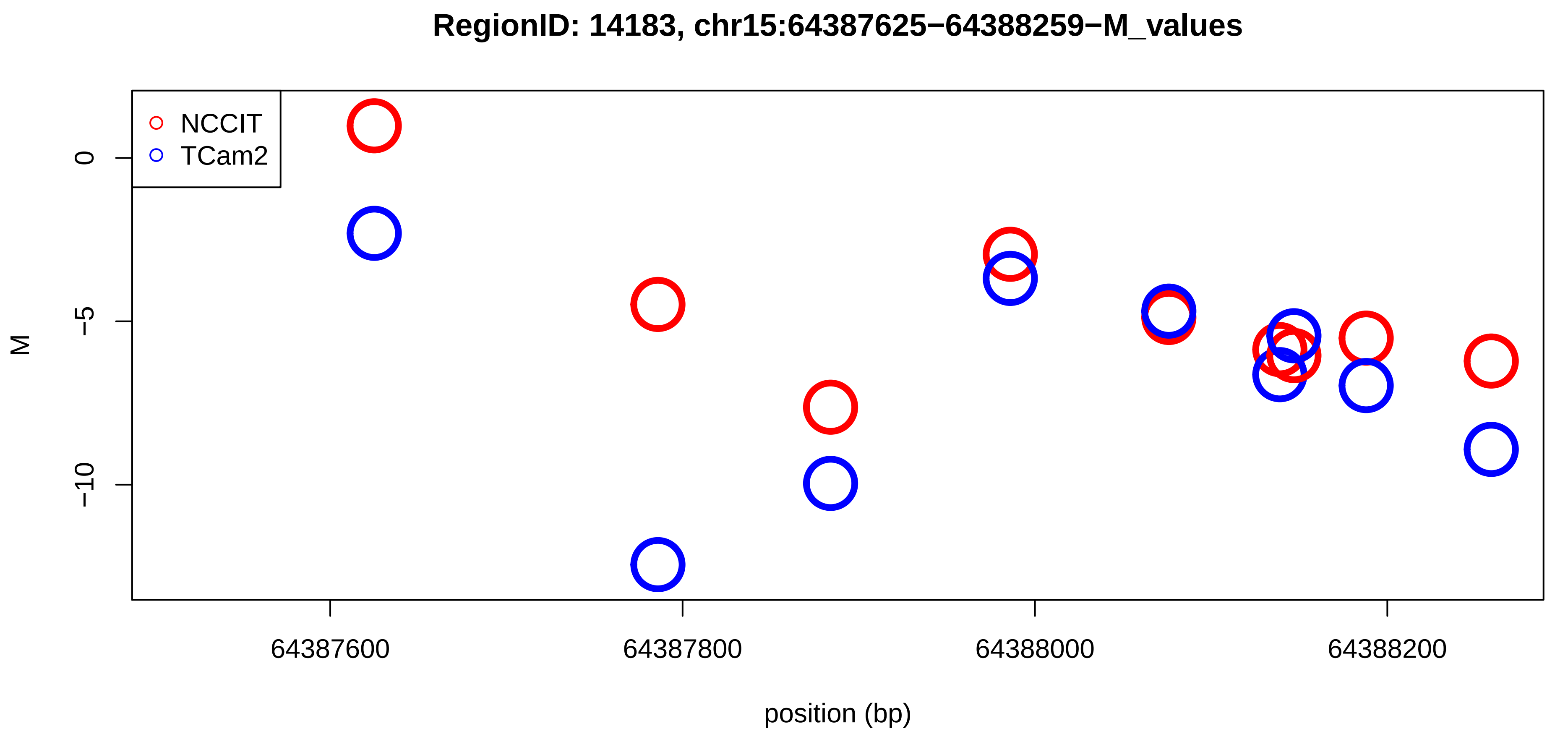
RegionID: 14183, chr15:64387625−64388259−M_values
NCCIT
TCam2
M
0
−5
−10
64387600
64387800
64388000
64388200
position (bp)

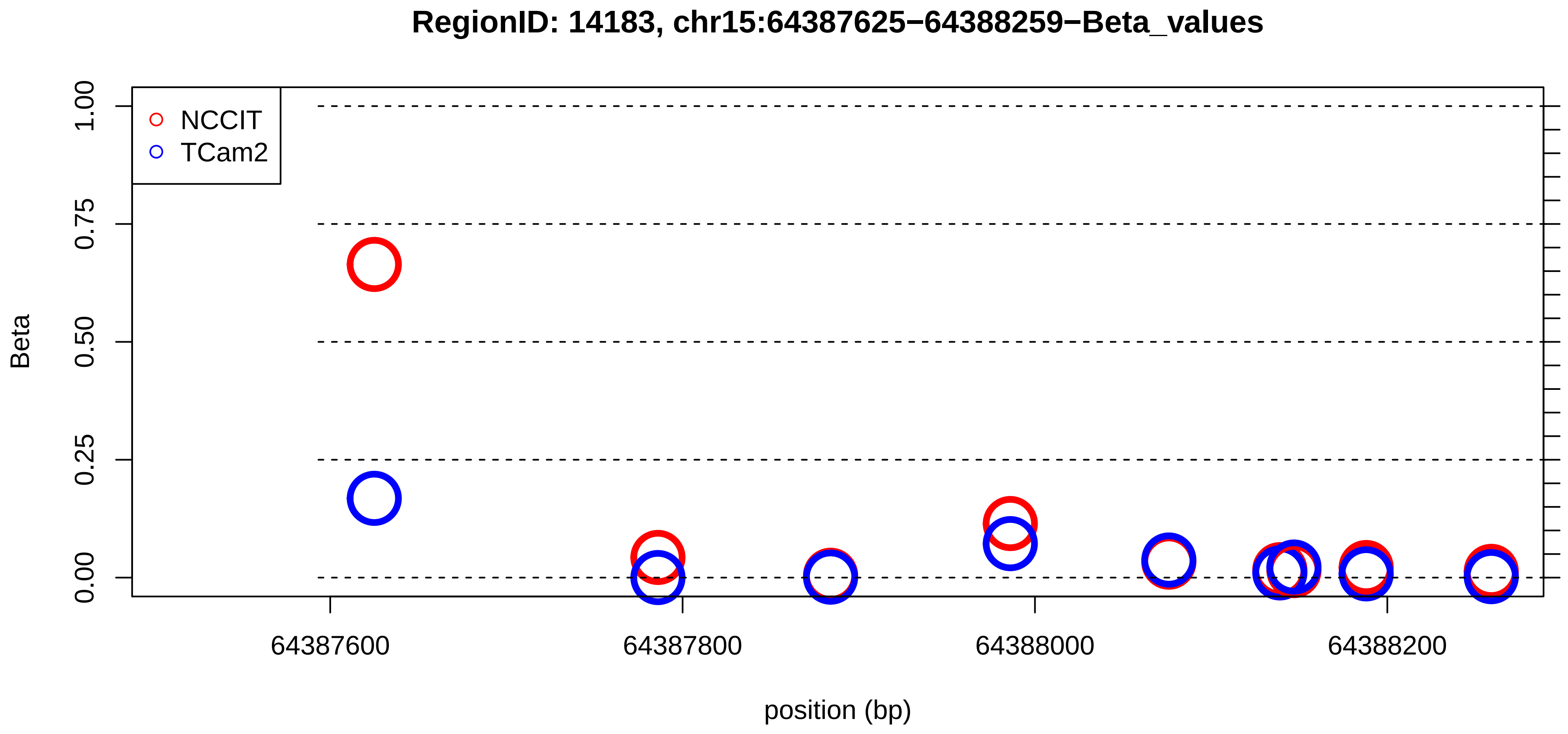
RegionID: 14183, chr15:64387625−64388259−Beta_values
NCCIT
TCam2
Beta
1.00
0.75
0.50
0.25
0.00
64387600
64387800
64388000
64388200
position (bp)
